# Hemoadsorption and plasma adsorption: two current options for the 3rd dimension of dialysis purification

**DOI:** 10.1007/s40620-025-02257-x

**Published:** 2025-03-12

**Authors:** Niccolò Morisi, Gaetano Alfano, Marco Ferrarini, Camilla Ferri, Francesco Fontana, Marco Ballestri, Gabriele Donati

**Affiliations:** 1 Nephrology Dialysis and Kidney Transplant Unit, Azienda Ospedaliero Universitaria di Modena, Via del pozzo 71, 41122 Modena, Italy; 2 Department CHIMOMO, University of Modena and Reggio Emilia, Modena, Italy

**Keywords:** Hemoadsorption, Plasma adsorption, HFR, PEPA, Uremic toxins

## Abstract

The adsorption technique has opened a new frontier in the field of purification through hemodialysis. This technique has proved to be effective in removing uremic toxins previously deemed inaccessible due to their size or charge, as well as to their molecular interactions with blood proteins. In this context, this review provides a detailed explanation of the role of Polyester-polymer alloy (PEPA®) membranes and hemodiafiltration with endogenous reinfusion. The polyester-polymer alloy membrane is a three-layered dialysis membrane with adsorbent properties, representing an innovative approach to engineering the filter membrane to achieve adsorbent characteristics while maintaining biocompatibility with blood. Haemodiafiltration with endogenous reinfusion is a technique where a first filter separates plasma water from blood, then plasma water is led to a sorbent cartridge. After contact with the sorbent, the purified plasma water reconstitutes the whole blood, which is led to a second filter where diffusion takes place. Both techniques are efficacious in removing middle-molecular weight molecules and protein-bound toxins, and have a role in reducing inflammation and erythropoietin resistance. However, the additional clinical advantage of adsorption is partially offset by lack of selectivity in the absorption process, potentially resulting in the loss of micro- and macronutrients or drugs. Therefore, further studies are encouraged to evaluate the impact of absorption on patient outcomes and clinical applicability in certain medical conditions.

**Graphical abstract:**

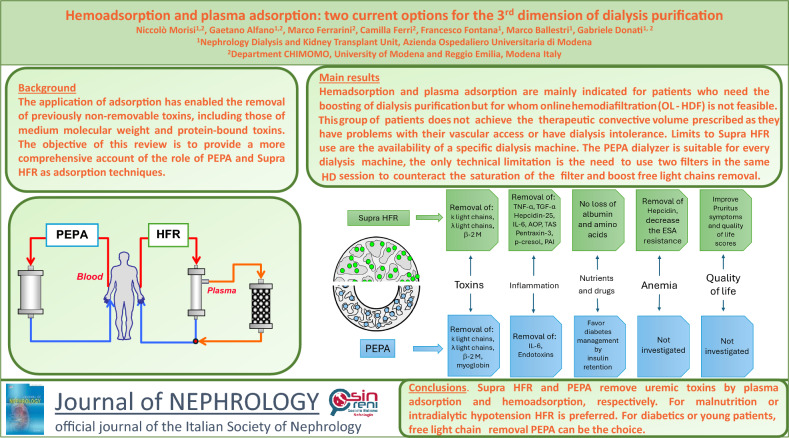

**Supplementary Information:**

The online version contains supplementary material available at 10.1007/s40620-025-02257-x.

## Introduction

The definition of “uremia” has changed as medical advances have been made, and is no longer associated with the classic triad of volume overload, hypocalcemia or anemia. Today, “uremia” refers to the accumulation of organic waste products, many still partially unidentified, that are normally removed by the kidneys [1]. According to mass action law [2], the biochemical effect on any toxin is linked to its concentration and depends on uremic generation and clearance. The actual concept of dialysis adequacy was based on urea and creatinine clearance [3].

In 2003, the European Uremic Toxins (EUTox) working group introduced a classification of uremic toxins based on the physicochemical properties influencing their clearance during conventional hemodialysis [4]:

- Small hydrophilic toxins: toxins under 500 Da (like Urea). Standard hemodialysis (HD) effectively removes them primarily using diffusion force [5];
- Medium-size hydrophilic toxins: toxins over 500 Da (like β2 microglobulin or parathyroid hormone). Convective transport can remove some of them but their size limits efficient clearance [6];
- Protein-bound toxins (PBUTs): toxins that exhibit more than 80% plasma protein binding. Despite their similar molecular weight, clearance is negatively affected by the lower concentration of unbound toxin [7].

Research on uremic toxins has progressed in the last decades. Indeed, recent studies on “-omic” sciences allow us to discover a great number of new retention solutes in uremia [5]. In the last years, adsorption systems have been studied to solve the limits of purification of diffusive and convective methods [8].

## The approach to sorbent technologies in clinical practice

At its core, adsorption involves the attachment of ions, atoms, or molecules from a gas or liquid onto the surface of a solid material, such as a sorbent. Unlike absorption, where the substance penetrates and is taken up into the interior of the material, adsorption is a surface-based phenomenon.

The binding of substances to a sorbent can occur through different physical or chemical mechanisms. Van der Waals forces, which arise from fluctuating dipole moments even in nonpolar molecules, play a significant role in this interaction. Additionally, hydrogen bonds, which form between a partially positive hydrogen atom and a partially negative atom on a neighboring molecule (with water being a common example), are crucial in many adsorption processes. Another important factor is hydrophobic interactions, where nonpolar substances are excluded from an aqueous environment, effectively binding to the adsorbent. These physical and chemical interactions collectively determine the efficiency and specificity of adsorption, making it a versatile and powerful tool in the field of medical treatments, particularly in the purification of blood or plasma during extracorporeal therapies. In order to use sorbents in clinical practice certain essential requirements must be met to ensure patient safety. The sorbent must be effective, biocompatible and safe for patients [9].

The use of sorbents for blood toxin removal has roots dating back nearly fifty years. The concept of hemoadsorption, where blood is brought into direct contact with an adsorbent material, was pioneered by Yatzidis and colleagues in 1967 [10]. Their initial studies primarily focused on treating barbiturate poisoning, demonstrating the potential of this technique in emergency toxicology. As research progressed, the scope of hemoadsorption expanded, and by 1978, scientists began exploring its application in managing hepatic insufficiency. Despite the promise of this technique, early clinical outcomes were often limited by significant adverse effects, which dampened its widespread adoption [11].

**Fig. 1:**
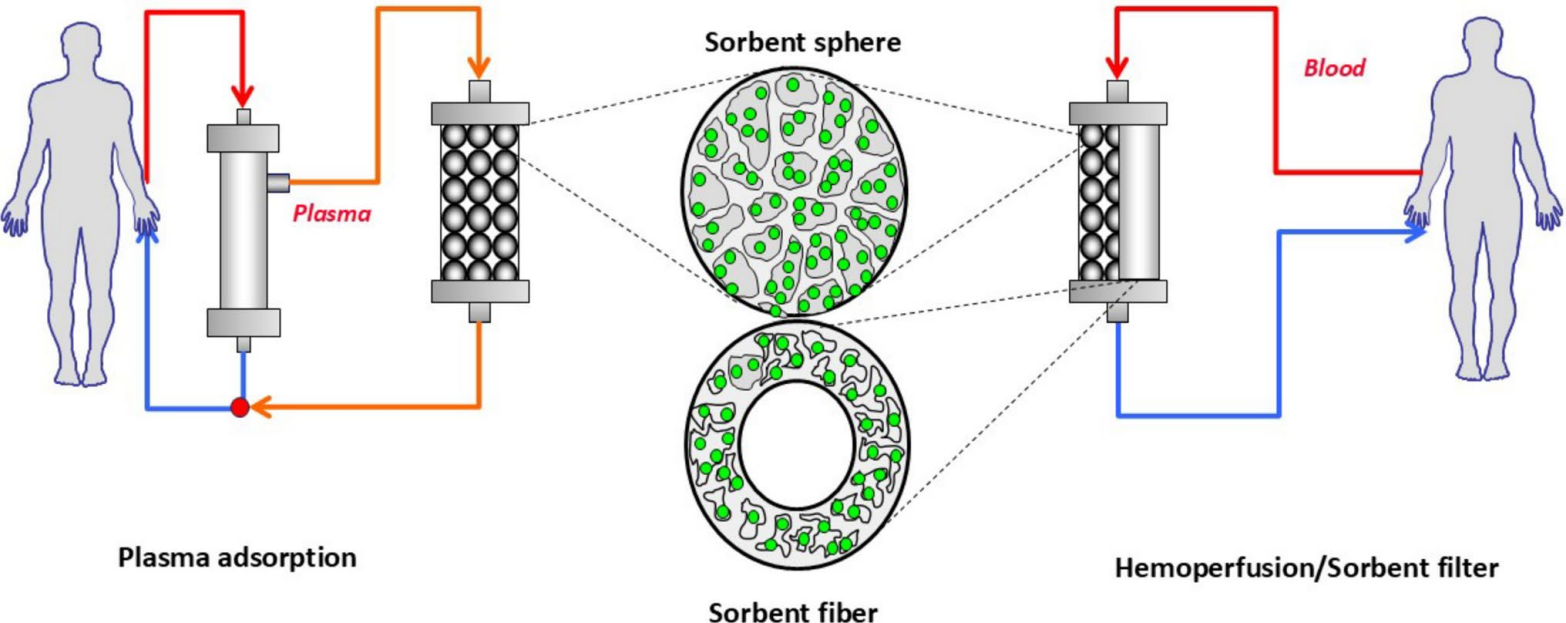
Different approaches to middle weight toxin clearance. The schematic illustrates two methods for improving the removal of medium-weight uremic toxins. Plasma filtration is shown on the left and hemoadsorption (with beads) or a classical dialysis scheme with a sorbent filter is shown on the right

In response to these challenges, clinicians and researchers have been actively developing methods to mitigate these adverse effects, leading to two principal approaches (see Fig. 1) [12]. The first focuses on physically separating the sorbent material from direct contact with biological fluids. This is achieved by coating the sorbent with various molecules, such as albumin, cellulose, or other biocompatible substances, which create a barrier that reduces the interaction between the sorbent and blood components, thereby minimizing potential side effects. This has led to two types of blood purification techniques: the first with sorbent beads, called hemoadsorption; the second using dialyzer membranes with different layers and properties. The latter approach involves a more complex procedure whereby the plasma is separated from the cellular components of the blood before hemoadsorption. This technique, known as plasma adsorption, allows for the selective purification of plasma, reducing the risk of damage to blood cells and other adverse events associated with direct hemoadsorption.

## Polyester polymer alloy membrane

**Fig. 2:**
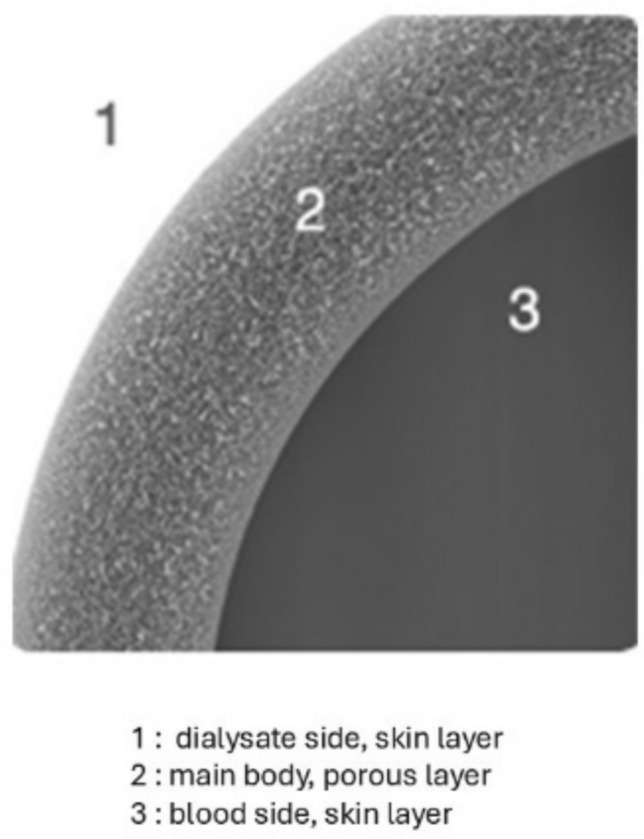
Morphologic structure of PEPA fibers. Picture capture by SEM (scanning electron microscope) where the three-layer structure of each fiber is well defined. Picture from Nikkiso

Following the first approach described above, many polymers have been studied to find the right balance between biocompatibility and sorption efficiency. The Polyester Polymer Alloy (PEPA) membrane was developed as an advanced synthetic polymer membrane that combines the benefits of multiple polymer components, and was first marketed by Nikkiso as FLX. Specifically, it is composed of two key polymers: polyethersulfone (PES) and polyacrylate (PAR). The special feature of this membrane is its ability to regulate pore size through precise manipulation of the blend ratio between polyethersulfone and polyacrylate, providing enhanced control over its filtration characteristics. One of the unique structural features of the polyester-polymer alloy membrane is its three-layer composition: it consists of an inner surface skin layer, an intermediate porous layer and an outer surface skin layer (see Fig. 2). This multi-layered architecture plays a critical role in its overall functionality. The inner surface skin layer regulates the permeability of water and various solutes, thereby contributing significantly to the membrane’s filtration performance. As such, the polyester-polymer alloy membrane dialyzer is regarded as a high-performance dialyzer, capable of efficiently facilitating dialysis by controlling selective permeability based on clinical needs. Furthermore, the skin layer that is present on the outer surface of the membrane offers an additional benefit by retaining molecules such as endotoxins, and has the hydrophilic capacity to retain molecules such as β 2 microglobulin by an additive amount of polyvinylpyrrolidone. All of these characteristics define a filter with sorbent properties  that can adapt to different clinical settings [13]. The hydrophilic polyester-polymer alloy membrane was marketed by Nikkiso in two different dialyzers with different water permeability: FDX and FDY. See Table 1 Supplementary Material for details.

Several studies have been conducted on dialysis filters made of polyester-polymer alloys. Specifically, regarding the removal of low-molecular-weight toxins, it has been demonstrated that the FDY filter is not inferior to the more traditional polysulfone filter. Regarding middle-molecular-weight toxins, the study by Maduell et al. [14] demonstrated that the FDY dialyzer, compared to the conventional polysulfone filter, showed a higher reduction ratio for free-light chains (polyester-polymer alloy reduction ratio 70% for k light chain and 72% for *λ* light chain) and myoglobin, while no significant differences were observed for β2 microglobulin. Belmuaz and colleagues determined that polyester-polymer alloy filters are highly beneficial when utilized in bicarbonate dialysis, given their classification as super high flux dialyzers with a β2 microglobulin sieving coefficient exceeding 0.7. Furthermore, a notable rate of free light chain removal was observed, without dosing free light chains in the spent dialysate. This confirmed the adsorption properties of the dialyzer [15]. Additionally, the work of Donati et al. indicated that there were no differences between the two types of hydrophilic polyester-polymer alloy filters (FDX and FDY) and between these filters and the PMMA BK-F membrane in the removal of free light chains [16]. There are not many studies on the impact of polyester-polymer alloy filters on inflammatory status. However, experiments on rats conducted by Hayata et al. [17] effectively demonstrated that the filter can absorb IL-6 within its layers. A similar phenomenon had already been observed concerning the filter’s ability to absorb endotoxins [18]. Finally, regarding nutrient loss through the filter, it has been confirmed that the polyester-polymer alloy membrane tends to result in greater losses. Poitier showed experimentally that an albumin leakage > 5g/session takes place when the convective flux adjusted for the body surface area exceeds 15 L/ session, with either the FDY or the FDX membrane [19]. In particular, it has been observed that the polyester-polymer alloy membrane has an albumin reduction ratio of around 10% compared to polysulfone. As a consequence, although albumin loss in the dialysate may be clinically acceptable during FDY treatments, it is important to use these dialyzers in HD mode [14] or hemodiafiltration (HDF) with low convective flux [16], until Potier’s study is disproved. Additionally, there is a greater loss of amino acids, with an overall loss close to 20% per dialysis session [20]. Ornithine, phenylalanine, tryptophan, and cystine are lost in greater quantities during hemodialysis with both hydrophilic and non-hydrophilic polyester-polymer alloy membranes. Careful monitoring of amino acid loss is essential to enhance nutritional management in HD patients [20]. Another characteristic of polyester-polymer alloy filters that has been overlooked is their membrane zeta potential, which is more negative than that of other new synthetic filters available on the EU market [21]. This characteristic enables the retention of insulin due to its negative charge, which may be beneficial during dialysis for diabetic patients dependent on insulin [22]. It is important to note that this zeta potential precludes the use of polyester-polymer alloy filters in patients with hypersensitivity reactions to synthetic dialyzers [21]. The polyester-polymer alloy dialyzer demonstrates a high capacity for the adsorption of nafamostat mesylate, with complete adsorption observed from the first to the seventh flow-through, and significant adsorption persisting up to the tenth flow-through. This necessitates careful dosing of nafamostat mesylate to mitigate the increased risk of circuit coagulation during treatment [23]. However, no coagulation-related complications have been reported in the various studies analyzed. Table 1 summarizes all studies cited.

**Table 1:** Study summary for polyester polymer alloy membranes

Country	References	Year	*N*° patients/*n*° centers	Membrane	Treatment modality	Main results
Uremic toxins						
Spain	Maduell et al. [14]	2024	21 patients/1	PEPA FDY and PS in HD	Standard HD for 4–5 h, Qb 450 mL/min, Qd 400 mL/min, 3 sessions/week	PEPA showed better RR of Myoglobin (74%), *k* (70%) and *λ* (72%) free light chains and prolactin (72%). No differences in β2M RR
Italy	Donati et al. [16]	2022	23 patients with MM/1	PEPA FDX versus PEPA FDY in ol-HDF versus PMMA in HD, all using two dialyzers per session	Standard HD and ol-HDF for 4 h, Qb 300 mL/min, Qd 500 mL/min, Cv 20 L, 3 sessions/week, all using two dialyzers per session	No differences in free light chain RR
Inflammation and oxidative stress						
Japan	Maeda et al. [17]	2017	Rat sepsis model/1	PEPA FLX vs CTA vs sham		PEPA filter in hemofiltration significantly adsorbed IL-6
Japan	Nakatani et al. [18]	2003	In vitro study	FDY and FLX PEPA		All membranes showed the capacity to adsorb endotoxins
Nutrition and albumin losses						
Spain	Maduell et al. [14]	2024	21 patients/1	PEPA FDY and Fx in HD	Standard HD for 4–5 h, Qb 450 mL/min, Qd 400 mL/min, 3 sessions/week	PEPA lost more albumin than Fx (10.1% versus 7.6%)
Italy	Donati et al. [16]	2022	23 AKI patients with MM/1	PEPA FDX versus PEPA FDY in ol-HDF versus PMMA in HD, all using two dialyzers per session	Standard HD and ol-HDF for 4 h, Qb 300 mL/min, Qd 500 mL/min, Cv 20 L, 3 sessions/week, all using two dialyzers per session	RR of albumin was similar between three filters (PMMA 15%, FDX-PEPA 10%, FDY-PEPA 9%)
Japan	Yokomatsu et al. [20]	2013	9 patients/1	FDX and FLX PEPA versus PAN in HD	Standard HD for 3–4 h, Qb 100–200 mL/min, Qd 500 mL/min, 3 sessions/week	The loss of ornithine, phenylalanine, tryptophan, and cystine were greater with hydrophilic and non-hydrophilic PEPA membranes compared to PAN membranes

The table summarizes the characteristics of the studies that evaluated PEPA’s performance in the different areas

*PEPA* polyester polymer alloy, *PS* polysulfone, *HD* hemodialysis, *ol-HDF* online hemodiafiltration, *RR* reduction ratio, *β2M* beta-2 microglobulin, *MM* multiple myeloma, *PMMA* polymethyl methacrylate, *CTA* cellulose triacetate, *IL-6* interleukin-6, *PAN* polyacrylonitrile

## Hemodiafiltration with endogenous reinfusion

**Fig. 3:**
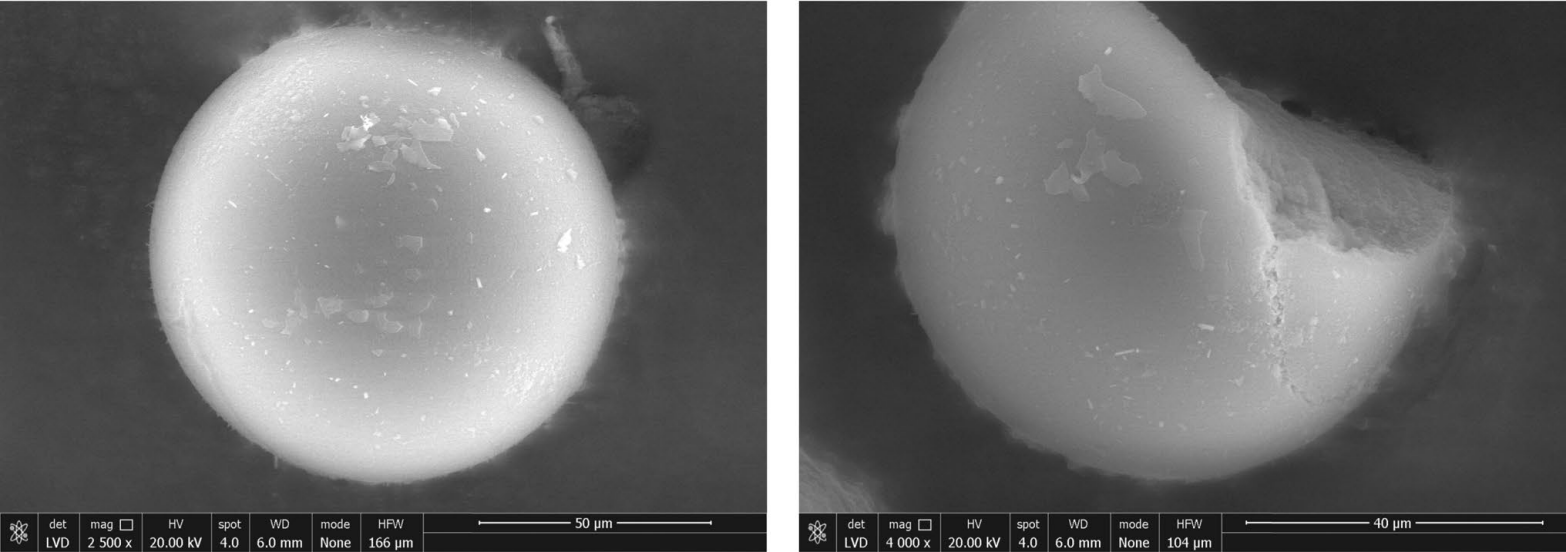
Morphologic structure of HFR styrenic resin. Picture capture by *SEM-FEG* scanning electron microscopy—field emission gun. (Centro interdipartimentale grandi strumenti UNIMORE. Nova NanoSEM 450, Fei Company–Bruker Corporation, Hillsboro OR/Waltham MA, USA 2013). *Det* the type of detector used, *Mag* magnification, *HV* electric current used to power the electron source, *Spot* the size of the electron beam, *WD* work distance between the sample and the field emission gun, *HFW* accelerating voltage. This is the electrical potential that accelerates the electrons

The development of hemodiafiltration with endogenous reinfusion directly correlates with the second approach described above and it began with early studies using a method known as paired filtration dialysis [24, 25]. This innovative technique was designed to enhance the efficacy of hemodialysis by incorporating sequential hemodiafiltration with ultrafiltrate treatment. In the paired filtration dialysis process, blood is first passed through a filter that performs convection, effectively extracting this plasma filtrate. This contains various solutes and middle molecules like β2 microglobulin. Early experiments with paired filtration dialysis demonstrated the method’s efficacy in removing β2 microglobulin, showing promise in enhancing the clearance of middle molecules, improving patient outcomes, and potentially reducing complications associated with traditional hemodialysis [26]. The evolution of hemodiafiltration with endogenous reinfusion (HFR) represents a significant advancement in blood purification techniques, building upon the principles established by paired filtration dialysis [27–29]. After being treated by a sorbent cartridge, the plasma filtrate in hemodiafiltration with endogenous reinfusion is reintroduced into the bloodstream before entering a second filter, which performs traditional dialysis. This sequential process allows for the efficient removal of a broad range of solutes by combining the convective removal of larger molecules and the selective adsorption of specific toxins. The development of hemodiafiltration with endogenous reinfusion was a complex and iterative process, marked by numerous advancements aimed at identifying a suitable adsorbent capable of retaining a wide variety of mediators while still remaining appropriate for extracorporeal treatment. The latest and most effective iteration of this technology, Supra-hemodiafiltration with endogenous reinfusion, reflects these efforts, offering enhanced performance and greater efficacy in blood purification [30]. A distinctive feature of hemodiafiltration with endogenous reinfusion is endogenous reinfusion, which has enabled a progressive increase in the permeability of the membrane used during the initial convective phase, surpassing the traditional limits set for albumin (Table 2 Supplementary Material). This is possible because the resin used in hemodiafiltration with endogenous reinfusion does not adsorb the albumin present in the ultrafiltrate, allowing it to be reinfused back into the patient’s bloodstream, see Fig. 1. In Supra-hemodiafiltration with endogenous reinfusion, this convective phase utilizes a super-high-flux membrane (Synclear 0.2®, Bellco/Medtronic) with a surface area of 0.7 m^2^ and a high cut-off value (45,000 Da), significantly exceeding that of standard high-flux membranes commonly employed in online hemodiafiltration (OL-HDF), Fig. 3. The membrane’s screening coefficient closely resembles that of the glomerulus, providing enhanced filtration efficiency. Compared to the high-flux polyphenylene membrane used in traditional hemodiafiltration with endogenous reinfusion, this advanced membrane allows the ultrafiltrate to contain significantly higher concentrations of medium-to-high molecular weight molecules, which can subsequently be adsorbed. These include molecules like IL-6 (24,700 Da), α1-glycoprotein (43,500 Da), and albumin (66,500 Da). The ultrafiltrate, enriched with these molecules is then passed through an 80 g neutral styrene resin cartridge with an extensive adsorption area of 35,000 m^2^, ensuring effective removal of targeted solutes before reinfusion [31, 32].

Various aspects of Supra-hemodiafiltration with endogenous reinfusion have been studied extensively. Regarding the removal of toxins, Donati et al. [33] confirmed that hemodiafiltration with endogenous reinfusion reduces free-light chains better than standard HD with a reduction ratio of 44% for k-light chains and a reduction ratio of 30% for *λ* light chains in chronic dialysis patients. These results have been confirmed by different studies [34]. The study by Murgia et al. [35] involving multiple myeloma patients confirmed the ability of Supra-hemodiafiltration with endogenous reinfusion to reduce highly concentrated light chains without loss of performance in patients with myeloma cast nephropathy. Another feature studied for Supra-hemodiafiltration with endogenous reinfusion was inflammation and oxidative status during treatment. In 2010, Calò et al. [28] demonstrated that hemodiafiltration with endogenous reinfusion treatment resulted in less oxidative stress by quantifying the expression of several mRNAs. Similar results were obtained with different markers of inflammation, such as advanced protein oxidation products, Tumor Necrosis factor α, interleukin 6, hepcidin-25, p-cresol, Indoxyl sulphate, proinflammatory monocytes CD14^+^/CD16^+^ and CD14^++^/CD16^+^ [28, 29, 31, 36–39]. There were few studies concerning the effect of this technique on anemia. The survey by Bolasco et al. [40] demonstrated that hemodiafiltration with endogenous reinfusion therapy reduces the need for erythropoietin stimulating agents. Later, in 2018, Tessitore et al. [36] discovered that Supra-hemodiafiltration with endogenous reinfusion reduced the level of hepcidin more than HD did. Nutritional status and quality of life were also studied. Interesting findings showed that the sorbents did not remove macronutrients, such as vitamins or albumin [35, 38, 41, 42]. Moreover, Supra-hemodiafiltration with endogenous reinfusion showed a great ability to reduce pruritus [43, 44] and enhance Quality of Life scores [45, 46]. In Table 2 we summarize the collected studies.

**Table 2:** Study summary for HFR and Supra-HFR

Country	References	Year	*N*° patients/*n*° centers	Technique	Treatment modality	Main results
Uremic toxins						
Italy	Murgia et al. [35]	2022	13/1	Supra-HFR in MM patients	3.30 h, Qb 250 mL/min, Qd 500 mL/min	Decrease free-light chain (RR for *k* 85%, for *λ* 45%) in ten sessions
Spain	Pendón-Ruiz de Mier et al. [34]	2020	9/1	Supra-HFR in MM patients	4 h, Qb 300–350 mL/min, Qd 500 mL/min; EU 13.2–14.4 L	Decrease free-light chain (RR for *k* 57%, for *λ* 44%) per session
Italy	Donati et al. [33]	2016	20/1	HFR vs. standard HD	4 h, Qb 310 mL/min, Qd 500 mL/min, EU rate 2.3 mL/h	Decrease free-light chain (RR for *k* 44%, for *λ* 30%). Decrease of β2M (RR 42%)
Inflammation and oxidative stress						
Italy	Donati et al. [31]	2022	9/1	Supra-HFR vs. OL-HDF	Qb 290 mL/min, Qd 500 mL/min, EU 13 L	Significant reduction of FGF23 with both techniques. IL-6 is not modified. Significant decrease of TNF-α and TGF-α with Supra-HFR, IL-8 is not modified
Italy	Tessitore et al. [36]	2018	28/1	Supra-HFR vs. low-flux HD	4 h, Qb 280 mL/min, Qd 500 mL/min	Significant decrease of TNF-α and Hepcidin-25 in HFR. A similar change in IL-6, CRP and Pentraxin-3 between methodics
Spain	Esquivias-Motta et al. [37]	2017	17/1	Supra-HFR vs. OL-HDF in non-inflamed patients		Decrease of all 13 biomarkers of inflammation as IL-6, TNF-α, the proportion of activated proinflammatory monocytes, p-Cs, IS
Italy	Palleschi et al. [38]	2016	41/19	Supra-HFR vs. OL-HDF	4 h, Qb > 300 mL/min, Qd 500 mL/min, EU 13L, CV 12L	Decrease in oxidative stress markers such as AOPP and TAS
Italy	Riccio et al. [39]	2014	12/11	Supra-HFR vs. OL-HDF in inflamed patients	4 h, Qb 330 mL/min, Qd 500 mL/min, EU rate 60 mL/min,	Increased adsorption of IL-6 and p-Cs on the Supra-HFR resin cartridge
Spain	González-Diez et al. [29]	2014	40/multicenter	Supra-HFR vs. standard HD in patients with ferritin < 600 μg/l		HFR show modest changes of oxidative parameters than HD
Italy	Calò et al. [28]	2010	14/6	HFR vs. standard HD in non-inflamed patients		Decrease in oxidative stress markers as OxLDL, PAI-1 mRNA and p22^phox^
Anemia						
Italy	Tessitore et al. [36]	2018	28/1	HFR vs. low-flux HD	4 h, Qb 280 mL/min, Qd 500 mL/min	Significant hepcidin decrease
Italy	Bolasco et al. [40]	2011	30/14	HFR vs. standard HD in non-inflamed patients	3.30–4 h, Qb 300–350 mL/min, Qd 500 mL/min, CV rate 3L/hour	Increased hemoglobin and decreased ESA requirements
Nutrition and albumin losses						
Italy	Murgia et al. [35]	2022	13/1	Supra-HFR	3.30 h, Qb 250 mL/min, Qd 500 mL/min	No loss of albumin per session
Italy	Palleschi et al. [38]	2016	41/19	Supra-HFR vs. HFR and OL-HDF	4 h, Qb > 300 mL/min, Qd 500 mL/min, EU 13L, CV 12L	Sorbent resins do not remove Vitamin C. Supra- HFR decreases RPB. No differences in Vitamin A and E
Italy	Borrelli et al. [41]	2014	24/8	HFR in inflamed patients	Qb 300 mL/min, Qd 500 ml/min, EU 11.9L	Increased albumin levels and no loss of amino acids
Italy	Borrelli et al. [42]	2010	48/10	HFR vs. AFB	Qb 300 mL/min, Qd 500 mL/min, CV 12 L, EU 11.9 L	No loss of amino acids
Quality of life						
China	Fei Zha et al. [43]	2023	60/1	Supra-HFR vs. standard HD	4 h, Qb 200–250 mL/min, Qd 500 mL/min	Improvement of pruritus
Italy	Scaparrotta et al. [44]	2021	1/1	Supra-HFR vs. standard HD		Improvement of pruritus and quality of life in a liver transplant patient
Italy	Solano et al. [45]	2015	1/1	Supra-HFR vs. standard HD		Improvement of RAND 36-Item Health Survey in a patient with lupus nephritis
Italy	Borrelli et al. [46]	2016	114/18	HFR vs. standard HD	Qb 300 ml/min, Qd 500 mL/min	Improvement of the physical component of SF-36

The table summarizes the characteristics of the studies that evaluated HFR and Supra-HFR performance in the different areas

*N* number, *HFR* hemodiafiltration with reinfusion of the endogenous ultrafiltrate, *MM* multiple myeloma, *RR* reduction ratio, *HD* hemodialysis, *β2M* beta-2 microglobulin, *ol-HFR* online hemodiafiltration, *FGF23* fibroblast growth factor 23, *IL* interleukin, *TNF-α* tumour necrosis factor α, *TGF-α* tumour growth factor α, *p-Cs* para-cresyl sulfate, *IS* indoxyl-sulphate, *AOPP* advanced oxidation protein products, *TAS* total antioxidant status, *OxLDL* oxidized low-density lipoprotein, *PAI-1* plasminogen activator inhibitor-1, *mRNA* messenger ribonucleic acid, *ESA* erythropoietin stimulating agents, *RPB* retinoid binding protein, *AFB* acetate free biofiltration, *SF-36* short form health survey 36

## Indications and limits

**Fig. 4:**
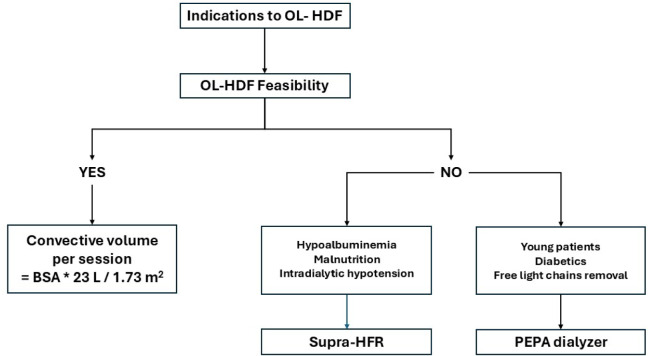
Flowchart of modality indications. The flow chart shows the indications for Supra-HFR or PEPA when it is not possible to perform OL-HDR. *OL-HDF* online hemodiafiltration, *BSA* body surface area, *HFR* hemodiafiltration with adsorption resin, *PEPA* polyester polymer alloy

Hemadsorption and plasma adsorption are mainly indicated when we need to boost dialysis purification but OL-HDF is not feasible. Locatelli et al. carried out an elegant survey among European nephrologists and showed the indications for OL-HDF prescription were restless legs syndrome, cardiovascular disease, pruritus, recent inflammatory state, erythropoietin stimulating agent resistance, and increased levels of middle molecular weight toxins. Nonetheless, there are a number of patients who are not suitable for OL-HDF [47]. This group of patients does not achieve the prescribed therapeutic convective volume since they have problems with vascular access or dialysis intolerance [48]. In such cases, Supra-hemodiafiltration with endogenous reinfusion or a polyester-polymer alloy dialyzer can be prescribed. The choice between the two depends on clinical and technical factors. The clinical basis for choosing Supra-hemodiafiltration with endogenous reinfusion includes hypoalbuminemia and malnutrition or intradialytic hypotension. In the former case, we can assure no albumin leakage during dialysis nor amino acid loss [41]. In the latter case, the biofeedback system called “Aequilibrium” can be applied to avoid intradialytic hypotension [49]. With regard to polyester-polymer alloy dialyzers, they should be used in young patients who need the boosting of dialysis purification due to erythropoietin stimulating agent resistance or an inflammatory state, in patients with diabetes undergoing insulin therapy [22], and in patients affected by acute kidney injury (AKI) due to multiple myeloma to remove free light chains with the use of two filters during each 4-h HD session [16]. Figure 4 summarizes the clinical indications in a flowchart.

A separate discussion is warranted regarding the application of these methods in acute settings. Notably, both hemodiafiltration with endogenous reinfusion and polyester-polymer membranes have demonstrated efficacy during the acute phase of treatment for multiple myeloma. For instance, Supra-hemodiafiltration with endogenous reinfusion has shown a significant capacity to reduce free light chains, with reduction ratios of 44% for κ light chains and 30% for *λ* light chains, as reported by Pendón-Ruiz de Mier et al. [34], and this effectiveness has been further confirmed in studies involving multiple myeloma patients by Murgia et al. [35]. Similarly, polyester-polymer alloy filters, particularly the FDY dialyzer, have been shown to achieve higher reduction ratios for light chains and myoglobin compared to conventional filters, making them valuable tools in managing acute conditions associated with multiple myeloma [14]. In addition, while hemodiafiltration with endogenous reinfusion has been effective in removing myoglobin during rhabdomyolysis AKI only in pilot studies [50, 51], there are still no studies that have demonstrated filter efficacy in polyester-polymer alloy membranes in this type of AKI.

Currently, no other acute applications have been thoroughly studied, but given their mechanisms of action, these technologies hold potential for use in scenarios such as cytokine storms (e.g., during sepsis) or in cases of drug/poison intoxication. Further research into these areas could broaden the therapeutic scope of hemodiafiltration with endogenous reinfusion and the use of polyester-polymer alloy membranes in acute clinical contexts [52].

The cost implications of Supra-hemodiafiltration with endogenous reinfusion and polyester-polymer alloy dialyzers highlight important considerations for their use. Supra-hemodiafiltration with endogenous reinfusion costs approximately €90 per session and is exclusively compatible with Bellco dialysis machines, limiting its accessibility and increasing implementation costs. However, its advanced features, such as enhanced toxin removal and improved inflammatory control, may justify the expense in select cases like multiple myeloma. In contrast, the polyester-polymer alloy dialyzer, at €25 per session, is a more cost-effective and versatile option as it is compatible with any dialysis machine. When two polyester-polymer alloy filters are used in a single session to boost toxin removal, the cost doubles to €50, narrowing the gap with Supra-hemodiafiltration with endogenous reinfusion but maintaining greater affordability overall. While Supra-hemodiafiltration with endogenous reinfusion is suited for advanced cases, the polyester-polymer alloy membrane offers a flexible and less expensive alternative, particularly in resource-limited settings or where the specific dialysis machines are unavailable.

## Limitations and future directions

A key limitation of this review is that many of the studies analyzed are based on relatively small patient populations, which makes it challenging to draw comparisons between them. This limitation is intrinsic to the field due to the subject matter being both relatively recent and highly specific; the heterogeneity of study designs further complicates the ability to draw definitive conclusions. However, by highlighting the current state of research and identifying knowledge gaps, it is hoped that this review will encourage researchers to further explore and expand investigations into this promising area. Increased research efforts, particularly through larger and more standardized clinical trials, will be crucial to better understand and optimize the use of hemodiafiltration with endogenous reinfusion and polyester-polymer alloy membranes in clinical practice.

## Conclusion

A key objective in hemodialysis research is to overcome the current limitations in purification by employing novel techniques. The polyester-polymer alloy dialyzer has demonstrated significant purification capacity and inflammatory modulation. Further studies are required to evaluate its impact on erythropoietin stimulating agent modulation and quality of life. In contrast, the Supra-hemodiafiltration with endogenous reinfusion technique has been the subject of extensive study and offers several advantages in terms of purification, inflammation control, erythropoietin stimulating agent requirements and quality of life. The current challenge is to understand the potential evolution of this technique, particularly in view of recent progress. In conclusion, the enhancement of toxin and inflammatory mediator clearance determined by these techniques represents a pivotal objective in the advancement of dialysis research. However, it is imperative to ensure that essential molecules, including nutrients and albumin, are not removed during treatment.

## Supplementary Information

Below is the link to the electronic supplementary material.

Supplementary file1 (DOCX 14 KB)

Supplementary file2 (DOCX 15 KB)

## Data Availability

Not applicable.
