# Supplementary material for: Hemoadsorption and plasma adsorption: two current options for the 3rd dimension of dialysis purification
Source: J Nephrol. 2025 Mar 12;38(3):845–57. doi: 10.1007/s40620-025-02257-x (PMC12165880; doi:10.1007/s40620-025-02257-x)
Supplement: Supplementary file 1 — Supplementary file1 (DOCX 14 KB) [file 40620_2025_2257_MOESM1_ESM.docx]

**Table 1 Supplementary Material: Summary characteristics of** **Polyester polymer alloy filters**. PEPA, Polyester polymer alloy; KUF, ultrafiltration coefficient; NA, not available; β2M, beta-2 microglobulin.

|  | **FDY** | **FDX** |
| --- | --- | --- |
| **Membrane** | PEPA | PEPA |
| **KUF (mL/h/mm Hg)** | 64 | 63 |
| **Cut-off (kDa)** | 32.6 | 30 |
| **Wall thickness** | 30 | 30 |
| **Internal diameter** | 210 | 210 |
| **Length of fibre (mm)** | 260 | 260 |
| **Sieving coefficient β2M** | 0.94 | NA |
| **Sieving coefficient myoglobin** | NA | NA |
| **Sieving coefficient albumin** | NA | NA |
| **Sterilization** | Gamma ray | Gamma ray |
