# Supplementary material for: Hemoadsorption and plasma adsorption: two current options for the 3rd dimension of dialysis purification
Source: J Nephrol. 2025 Mar 12;38(3):845–57. doi: 10.1007/s40620-025-02257-x (PMC12165880; doi:10.1007/s40620-025-02257-x)
Supplement: Supplementary file 2 — Supplementary file2 (DOCX 15 KB) [file 40620_2025_2257_MOESM2_ESM.docx]

**Table 2 Supplementary Material: Summary characteristics of HFR and Supra-HFR.** The following table provides a summary of the principal characteristics of the Supra-HFR dialysis technique and of its precursor, HFR. HFR, Hemodiafiltration with Adsorption Resin; HF, Hemofiltration; HD, hemodialysis; KUF, ultrafiltration coefficient.

|  | **HFR** | | | **Supra-HFR** | | |
| --- | --- | --- | --- | --- | --- | --- |
|  | **HF** | **Sorbent** | **HD** | **HF** | **Sorbent** | **HD** |
| **Membrane** | **Polyphenylene LF** | **Styrene divinyl benzene (Selecta Plus) ®** | **Polyphenylene LF** | **Synclear 02** | **Styrene divinyl benzene (Suprasorb)®** | **Polyphenylene LF** |
| Surface area (m)^2^ | 0.7 | 700 m^2^ /g | 1.3/1.7 | 0.7 | 700 m^2^ /g | 1.3/17 |
| Cut-off (KDa) | 25 |  | 10 | 45 |  | 10 |
| KUF (ml/h*mmHg) | 28 |  | 10/13 | 36 |  | 10/13 |
| Volume (ml) |  | 43 |  |  | 80 |  |
| Sieving coefficient for albumin | 0.02 |  |  | 0.2 |  |  |
